# Mental health problems in Austrian adolescents: a nationwide, two-stage epidemiological study applying DSM-5 criteria

**DOI:** 10.1007/s00787-017-0999-6

**Published:** 2017-05-24

**Authors:** Gudrun Wagner, Michael Zeiler, Karin Waldherr, Julia Philipp, Stefanie Truttmann, Wolfgang Dür, Janet L. Treasure, Andreas F. K. Karwautz

**Affiliations:** 1 Department for Child and Adolescent Psychiatry, Medical University of Vienna, Waehringer Guertel 18-20, 1090 Vienna, Austria; 2 Ludwig Boltzmann Institute Health Promotion Research, Untere Donaustraße 47/3, 1020 Vienna, Austria; 3 FernFH Distance - Learning University of Applied Sciences, Zulingergasse 4, 3200 Wiener Neustadt, Austria; 4 Institute of Psychiatry, Psychology and Neuroscience, Kings College University, London, UK

**Keywords:** Mental disorders, DSM-5, Adolescents, Epidemiology

## Abstract

This is a nationwide epidemiological study using DSM-5 criteria to assess the prevalence of mental disorders in a large sample of Austrian adolescents between 10 and 18 years including hard-to-reach samples. A sample of 3615 adolescents from four cohorts (school grades 5, 7, 9, 11; age range 10–18 years) was recruited from 261 schools, samples of unemployed adolescents (*n* = 39) and adolescents from mental health institutions (*n* = 137) were added. The Youth Self-Report and SCOFF were used to screen for mental health problems. In a second phase, the Childrens’ Diagnostic Interview for Mental Disorders was used to make point and lifetime psychiatric diagnoses. Mental health service use was also assessed. Point prevalence and lifetime prevalence rates for at least one psychiatric disorder were 23.9% and 35.8%. The highest lifetime prevalence rates were found for anxiety disorders (15.6%), neurodevelopmental disorders (9.3%; ADHD 5.2%) and depressive disorders (6.2%). Forty-seven percent of adolescents with a lifetime psychiatric disorder had a second diagnosis. Internalising disorders were more prevalent in girls, while neurodevelopmental disorders and disruptive, impulse control and conduct disorders were more prevalent in boys. Of those with a lifetime psychiatric disorder, 47.5% had contacted mental health services. Of the residual 52.5% who had not contacted mental health services, 18.1% expressed an interest in treatment. DSM-5 mental health disorders are highly prevalent among Austrian adolescents. Over 50% had or were interested in accessing treatment. Early access to effective interventions for these problems is needed to reduce burden due to mental health disorders.

**Electronic supplementary material:**

The online version of this article (doi:10.1007/s00787-017-0999-6) contains supplementary material, which is available to authorized users.

## Introduction

Epidemiological studies are essential not only to provide data on children and adolescents who are affected by mental health problems, but also to provide information on the need, availability and access to mental health services. Information about prevalence and incidence is useful for planning primary, secondary and tertiary prevention and treatment services [1].

A meta-analysis of population studies assessing the prevalence of mental disorders in children and adolescents found a worldwide pooled prevalence rate of 13.4% for any mental disorder, surveys including epidemiological studies from 1970 to 2000 found median prevalence rates of 18%, and a US sample in 2010 revealed 20% of adolescents between 13 and 18 years with mental health problems who need treatment [2–7]. The most frequent psychiatric disorders in childhood and adolescence are anxiety disorders (up to 31.9%), behaviour disorders (16.3–19.1%), substance use disorders (8.3–11.4%), emotional disorders (3.7–14.3%), hyperkinetic disorders (2.2–8.6%) and aggressive dissocial disorders (2.1–7.6%) [2]. In childhood, mental health problems in general are more frequent in boys than in girls (2:1), but from the age of 13 years onward, the prevalence rates are higher for girls. However, there are gender differences between various disorders. For example, adolescent boys suffer more often from externalising and substance use disorders, while female adolescents are more often affected by internalising disorders, such as eating disorders and depressive disorders [3].

The severity and persistence of adolescent mental health problems are also of relevance [8–11]. In the Dutch TRAILS report, 22% out of 45% youth with a lifetime mental health disorder showed severe impairment. Functional impairment can be assessed from disorder-specific measures or a global measure such as the CGAS (Child Global Assessment Scale) [12]. Although anxiety disorders are most prevalent they are usually less severe, whereas the less prevalent mood and behaviour disorders are more severe [11]. The longitudinal BELLA study in Germany revealed a high persistence rate of mental health problems (>30% in a 6 year follow-up). Mental health service use was limited to only 33% with acute or recurrent mental health problems and 63.9% with persistent mental health problems [13]. A similar pattern was identified in the Dutch TRAILS cohort study with only 35% of adolescents with problems accessing specialised mental health services, increasing to 54.5% of those with multiple psychiatric disorders [10]. Of the 6–16 years old children and adolescents who were treated in specialised mental health services in Denmark, neurodevelopmental disorders and conduct disorders were the principal diagnostic group, especially in male children and adolescents, while affective, eating, neurotic, stress-related and adjustment disorders were more common in girls [8].

Several methodological issues are of relevance for the conduct of epidemiological studies. Information from multiple sources produces a more complete picture of behaviour and functioning [14]. The combination of a population-based cohort with a clinical cohort has been strongly recommended to examine continuities between the milder and the more severe ends of pathology. Barkmann and Schulte-Markwort (2004) suggest that additional quotas should be sampled from hard-to-reach groups such as inhabitants of youth welfare institutions or the offspring of migrants [15]. In Austria, there is compulsory education for nine school years, typically until the age of 15 years. According to surveys in the German-speaking area, about 5% are school refusers and 7.2% leave after 9 years [16]. These individuals who will not be captured from school surveys might overlap with those in mental health institutions or from institutions providing courses for adolescents not in education or employment.

The aim of our study was to determine the point and lifetime prevalence rates of psychiatric disorders (applying DSM-5 criteria) in a national school sample of Austrian adolescents aged between 10 and 18 years using a two-stage design which also sampled hard-to-reach groups.

## Methods

### Study design

The Mental Health in Austrian Teenagers (MHAT) study is a two-stage cross-sectional epidemiological study assessing mental disorders in a population of Austrian teenagers aged between 10 and 18 years. A random sample of adolescents from four age cohorts, grades 5, 7, 9 and 11 is drawn from different types of schools in all nine federal states of Austria (school sample). Additionally, adolescents not in school (early school leavers or school absenteeism) were also recruited through courses for unemployed youths and through mental health service centres. The latter formed the non-school sample.

A screening phase using questionnaires was followed by a diagnostic interview conducted by telephone applying the new criteria of the 5th edition of the Diagnostic and Statistical Manual of Mental Disorders (DSM-5, [17]). All adolescents scoring above a pre-determined cut-off point were recruited for detailed clinical interviews (“at-risk group”). Additionally, 10% of the adolescents scoring below the cutoff point were selected randomly for the clinical interview (“not at-risk group”). In addition to the adolescents, their parents (either mother or father) were also asked to participate in a clinical interview. The MHAT study was conducted between 2013 and 2015.

### Sample size calculation

A clustered prevalence sampling calculation based on Sullivan (2010) was used to obtain the required sample size for the screening stage [18]. For the sample size calculation, a prevalence of 20% of any psychiatric disorder in childhood and adolescence is used as a conservative assumption. Furthermore, a cluster size of 60 per school grade was intended and a design effect of 2 was assumed. The calculated sample size was *N* = 502 per school grade (in total *N* = 2008 for all four included school grades). As we also intended to calculate gender prevalence estimates without losing precision, the required sample size was doubled leading to a total required sample size of *N* = 4016. According to the national report on education, 7.2% of adolescents leave school after the compulsory 9 years (grade 7 and above) [16].  4% of the sample of 11th graders were recruited from training courses for early school leavers and unemployed adolescents (N = 40, n = 20 per sex). There might be some overlap with the group of adolescents who cannot attend a school due to severe mental health problems, children and adolescents with a psychiatric disorder who are often absent from school [19] approximate to 3% (*N* = 128, *n* = 16 per sex and school grade). Therefore, a sample of these adolescents was recruited from mental health service institutions (departments for child and adolescent psychiatry) in Austria.

A detailed description of sample size calculation is being published elsewhere [20].

### Procedure

Ethical approval for the study was obtained from the Ethics Commission of the Medical University of Vienna (EK 1134/2013). A multidisciplinary commission of the Austrian Federal Ministry of Education and Women’s Affairs also approved the study. For the screening phase, all Austrian schools (*N* = 2547) at lower and upper secondary educational level for pupils in the age group between 10 and 18 years were contacted by e-mail and/or telephone and invited to participate. Subsequently, a stratified random cluster sample was ascertained. School classes were recruited as distinct clusters, from strata defined by school type and federal state. Four school grades (5th, 7th, 9th and 11th) were included in the sample. No more than a maximum of two classes per school were selected. Individual exclusion criteria included low intellectual ability, low essential German language skills or attending a special needs school. Written informed consent from the adolescents and their legal representatives was obtained by the class teachers. Teachers rated all participating and non-participating students regarding school performance, behavioural problems and social integration. Adolescents completed the questionnaires during one school lesson (50 min). An online form and an equivalent paper–pencil form of the questionnaire were used (76.7% online). The feasibility and acceptability of the screening phase procedure was evaluated in a pilot study [21].

For the sample of early school leavers, different course providers for unemployed adolescents were selected randomly from a list of course providers and asked for participation. For the sample of mental health institutions, eight departments for child and adolescent psychiatry (out of 11) located in five federal states of Austria (in the following sections named as “clinical sample”) were selected and patients were asked to participate. The same procedure was applied as in the school sample with the difference that the paper–pencil version of the screening questionnaire was used only.

**Fig. 1:**
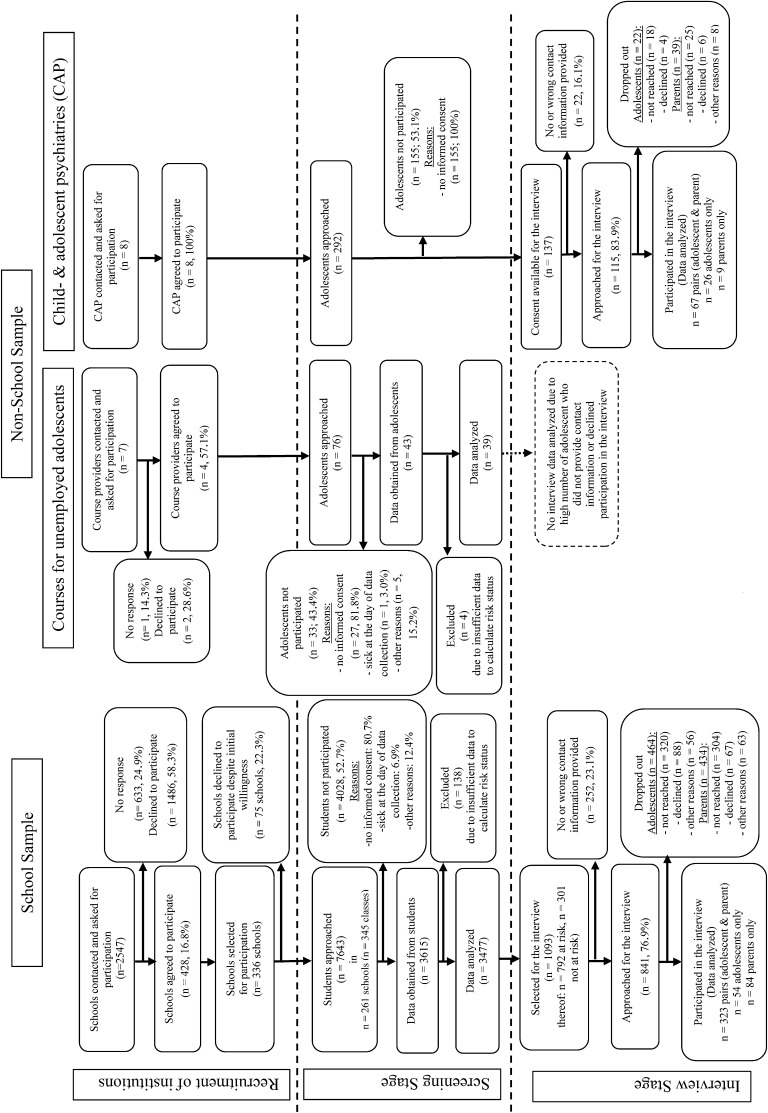
Flow diagram of recruitment process, screening and interview stage

The flow of the entire recruitment process is depicted in Fig. 1.

### Participants

With regard to the school sample, we approached 7643 adolescents. According to the teachers’ records, no student had to be excluded due to low intellectual ability and *n* = 12 had to be excluded due to low German language skills. We received questionnaires from a total of 3615 adolescents which correspond to a participation rate of 47.3%. One hundred and thirty-eight datasets had to be excluded because of too many missing values to calculate the risk status, leading to a final sample size of 3477 adolescents (44.7% males, 55.3% females).

To detect potential differences between participating and non-participating students, teachers rated all students of their class on a few basic questions. Logistic regression analyses were performed to predict participation (yes vs. no). According to the teachers, students who did not participate were more often absent from schools (*p* = 0.001), concentrated poorly (*p* = 0.001), were less socially integrated (*p* < 0.001), more socially withdrawn at school (*p* = 0.050) and had more behavioural problems (*p* = 0.040). However, effect sizes were all minimal to low (odds ratios 1.3–1.6).

In the sample of the unemployed adolescents and early school leavers, *n* = 76 adolescents were approached and data from 19 (48.7%) girls and 20 (51.3%) boys were obtained, which is approximately the planned sample size.

In the clinical sample, *n* = 292 patients aged 10–18 years were approached, of whom 137 (30 boys, 21.9% and 107 girls, 78.1%) provided informed consent for participation in the study.

### Screening (phase 1)

At the screening phase, socio-demographic data, mental health and potential risk factors affecting mental health were assessed by questionnaires including the following:

Socio-demographic variables include age, sex, school grade, school type, federal state, familial factors such as family structure (including single parent structure, stepparent in household), chronic somatic or mental diseases of parents and siblings, negative life events (death of close others, accident, traumatic experiences like physical and sexual abuse) and potential social risk factors such as migration background or low socio-economic status (including parental unemployment). The socio-economic status was assessed by the Family Affluence Scale [22].

The German version of the widely used Youth Self-Report was used to obtain the general psychopathology [23–25]. Behavioural and emotional problems were assessed by 103 problem items on a three-point scale (“0” = absent, “1” = sometimes true, “2” = often true). Items were summed up to eight syndrome scales including Withdrawn, Somatic Complaints, Anxious/Depressed, Social Problems, Thought Problems, Attention Problems, Delinquent Behaviour and Aggressive Behaviour, and three second-order scales (Total Problem score, Internalising Problems, Externalising Problems). Good internal consistencies were reported for the second-order scales (Cronbach alphas >0.86) and have been identified for the syndrome scales Anxious/Depressed and Aggressive Behaviour. They range between α = 0.80 and α = 0.86 for boys and girls. Internal consistencies for Somatic Complaints, Delinquent Behaviour and Attention Problems range between α = 0.70 and α = 0.77 and are satisfactory. Consistencies lower than α = 0.70 have been found for Withdrawn, and Thought Problems in boys and girls as well as Social Problems in boys [25].

Raw scores were transformed into T-scores according to existing German normative data.

As eating pathology is not covered in the Youth Self-Report, the SCOFF questionnaire [26] was used to assess a potential risk for eating disorders. The SCOFF is an acronym for the following five questions: Do you make yourself Sick because you feel uncomfortably full? Do you worry that you have lost Control over how much you eat? Have you recently lost more than One stone (6 kg) in a 3-month period? Do you believe yourself to be Fat when others say you are too thin? Would you say that Food dominates your life? The questions are answered on a dichotomous scale (“yes” vs. “no”). Those five items assess core features of anorexia nervosa, bulimia nervosa and binge eating disorders. One point is given for every “yes” answer. A score of ≥2 reflects a risk for an ED. A pooled sensitivity of 0.80 and a pooled specificity of 0.93 have been found in a meta-analysis of diagnostic accuracy of the SCOFF across several countries [27]. However, other disorders have not been included in the screening as they are better assessed through parent rating instead of self-rating, such as autism spectrum disorder. Following the completion of the screening questionnaire, the adolescents were divided into two groups (at-risk vs. not at-risk for mental disorders) according to pre-determined cut-offs derived from the Youth Self-Report and the SCOFF. The at-risk group was defined as reaching t-levels >70 in at least one of the eight YSR syndrome scales or a SCOFF total score of ≥2, including either weight loss of at least 6 kg within 3 months or intentional vomiting. All other adolescents who did not fulfil those criteria were assigned to the group “not at-risk”.

### Population at-risk

In the school sample, *n* = 792 (22.8%) reached the cut-off level, including *n* = 464 girls (24.1%) and *n* = 328 boys (21.2%) with 11 missing values in the variable sex and *n* = 2685 (77.2%) ranged below cut-off. Within the sample who scored over the cut-off level, *n* = 602 (76.0%) provided a telephone number and contact address for the telephone interview. We randomly selected *n* = 301 (11.2%) adolescents from the pool under the cut-off level; of those *n* = 62 (20.6%) did not provide a telephone number. Of those with contact address, in *n* = 323 cases, both one parent and the adolescent could be interviewed; in *n* = 54 cases only the adolescent and in *n* = 84 cases only the parent could be interviewed. In total, for *n* = 461 cases at least the adolescent’s or parent’s interview was available, leading to a total response rate of 54.8% of those who were approached. Those students who were selected for the interview but did not participate did not differ significantly from students who participated with regard to the YSR total score (mean = 47.32 vs. 48.79; *t* = 0.988, *p* = 0.323). In the sample of unemployed adolescents, *n* = 11 (57.9%) of the girls and *n* = 8 (40%) of the boys reached the cut-off level. In this group *n* = 21 (53.8%) did not provide contact information and *n* = 13 (33.3%) were not approachable for the interview or declined after initial consent. As from the remaining five unemployed adolescents no prevalence estimates are possible, we had to refrain from performing detailed interviews. Of the cases in the clinical sample, *n* = 115 (83.9%) provided contact information to approach them for the interview.

### Structured interview (phase 2)

All adolescents and respective parents selected for phase 2 underwent a structured clinical telephone interview (Childrens’ Diagnostic Interview for Mental Disorders; CDI-MD) [28].

The Childrens’ Diagnostic Interview for Mental Disorders (CDI-MD) is a structured clinical interview for children and adolescents aged from 6 to 18 years and their parents for assessing a broad range of psychiatric disorders. It comprises an interview guide for children and one for parents. The current published CDI-MD version for the diagnostics of psychiatric disorders according to the classification of DSM-IV-TR and ICD-10 has been adapted for the classification of DSM-5 by the authors of the CDI-MD. Point prevalence and lifetime prevalence rates were assessed for the following disorders:

Neurodevelopmental disorders [attention-deficit/hyperactivity disorder (ADHD), tic disorders], depressive disorders (disruptive mood dysregulation disorder, major depressive disorder), anxiety disorders (separation anxiety disorder, selective mutism, specific phobia, social anxiety disorder, panic disorder, agoraphobia, generalized anxiety disorder), obsessive–compulsive disorder, posttraumatic stress disorder, feeding and eating disorders (pica, anorexia nervosa, bulimia nervosa, binge eating disorder), elimination disorders (enuresis, encopresis), disruptive, impulse control, and conduct disorders (oppositional defiant disorder, conduct disorders). Listed under the section “Conditions for further study” in the DSM-5, suicidal behaviour disorder and non-suicidal self-injury can also be diagnosed by means of the CDI-MD. Screening questions for alcohol-, tobacco- and other substance-related disorders as well as for the schizophrenia spectrum and other psychotic disorders are included. The interview guides for assessing disrupted mood dysregulation disorder, suicidal behaviour disorder and non-suicidal self-injury have been included in the CDI-MD by Schneider and colleagues for the first time. Additionally, we have developed interview guides for the assessment of Internet gaming disorder (also in the “Conditions for further study”) as well as for avoidant/restrictive food intake disorder and rumination disorders.

The interrater reliability (kappa coefficient) ranged between 0.67 and 0.90 for the classes of lifetime diagnoses included in the children version and between 0.85 and 0.94 in the parent version [28]. Content validity can be derived from the classification scheme of the DSM-5. It is a widely used and well-accepted instrument for the assessment of mental disorders in children and adolescents.

The progression of the questions is syndrome oriented with skipping rules if the first two starting questions are not applicable. The prevalence of full-syndrome disorders will be presented in this paper. Full syndrome means that all diagnostic criteria of a psychiatric diagnosis were met, including subjective impairment. In the CDI-MD, impairment is judged by the interviewer on a four-point Likert scale from 0 = not at all to 3 = very strong and has to be at least 2 (= strong impairment) to be rated as clinical case. Constraint in four social areas (at home, at school or work, at leisure time, with friends) and personal suffering of the adolescent are considered. Evaluation as impairment is dependent on the individual diagnosis and follows the rules of DSM-5. If one diagnostic criterion was not reached but there was a significant impairment, a diagnosis within the category “other specified disorder” was assigned.

Furthermore, there are sections on the family history of mental disorders, as well as axis IV (psychosocial and environmental problems) and axis V (Global Assessment Functioning scale, ranging from 1 to 100). We chose to use the GAF score from DSM-IV representing the judgement of the individual’s overall level of functioning ranging from 1 to 100 (indicating the lowest 1 to the highest 100 levels of functioning) rather than WHO Disability Assessment Schedule (WHODAS) which has replaced it in DSM-5, to get the clinician’s judgement.

Interviewers were eight psychologists trained in clinical and health psychology, and one medical doctor in training for child and adolescent psychiatry. All interviewers completed a standardized training given by GW. Cases were discussed in regular supervision group meetings led by GW and KW to ensure a consistent approach across all interviewers. Each uncertain diagnosis was discussed intensively and a diagnosis was confirmed only when there was consensus.

For the present study, the interview sections were divided between adolescents and parents. Internalising disorders were assessed in the adolescents’ interview, and externalising disorders as well as disorders primarily occurring in infancy and early childhood were assessed in the parents’ interview. This decision was based on economic reasons and previous results which showed that externalising disorders can be observed and better judged by parents, whereas internalising disorders such as anxiety and depressive disorders cannot be identified well externally and are therefore better judged by the adolescents (see also [28]). Mental health service use was assessed by the end of each interview section by asking if any health service was used with regard to the reported problem and, if so, in what form (i.e. psychotherapy, psychological treatment, outpatient/inpatient treatment in a psychiatric clinic). Furthermore, the participants were asked if they had to take any medications due to the reported problem.

### Statistical analyses

Analyses were conducted using IBM Statistics 22.0 software and Microsoft Excel 2010. Data from the screening stage (including calculation of the risk status) were only analysed if there were no more than eight missing values in the YSR and no missing value in the SCOFF, which is in line with the manuals. The prevalence estimates of the psychiatric disorders obtained in the interview stage were derived as follows: for the school sample, prevalence estimates were calculated applying the law of total probability meaning that the prevalence estimates within the “at-risk” sample and within the “not at-risk” sample were pooled by weighting them with the probability for being at risk, respectively, and not at-risk. Prevalence estimates are provided separately for the “at-risk” and “not at-risk” group in a supplement table. Standard errors and confidence intervals were based on a simple random sampling. As there was a very high number of clusters (345 school classes) in combination with a low number of individuals within one cluster, standard errors calculated based on cluster sampling were quite the same as standard errors calculated based on a simple random sampling (design effect ~1).

The prevalence estimates in the clinical sample were calculated by the number of patients with a specific diagnosis divided by the total number of clinical patients who participated in the interview stage. As described below in more detail, the prevalence estimates within the sample of unemployed adolescents could not have been calculated due to the high dropout from the screening to the interview stage.

Finally, the prevalence estimates from the school and clinical sample were pooled to obtain a “total prevalence” that takes into account that a certain proportion of adolescents could not be reached via the school setting due to severe mental health problems. The school and clinical sample were weighted in accordance with the original sample plan. Detailed information on the calculations of the prevalence estimates can be requested from the corresponding authors.

The prevalence estimates for different psychiatric diagnoses are based on different numbers of cases. For the diagnoses that were assessed through the adolescent’s interview, prevalence estimates are based on all available adolescents’ interviews regardless of whether the parent’s interview was available or not. For the diagnoses that were assessed through the parents’ interview, prevalence estimates are based on all available parents’ interviews regardless of whether the adolescent’s interview was available or not. For calculating the prevalence of groups of psychiatric disorders (e.g. anxiety disorders) and the prevalence of any psychiatric disorder, all cases where both the adolescent’s and the parent’s interview were available were included. For all assessed diagnoses, the point prevalence and lifetime prevalence were calculated. The same procedure was applied separately to girls and boys to obtain gender-specific prevalence estimates. Furthermore, comorbidities between groups of psychiatric disorders were calculated. Only cases where both the adolescent’s and parent’s interview were available were included. Socio-demographic differences between students with and without a full-syndrome psychiatric diagnosis and the clinical sample were analysed by means of ANOVAs in case of metric variables and *χ* ^2^-tests in case of frequency variables. All factors that turned out as significant in the univariate analyses were further included in a multinomial logistic regression analysis to find out which factors still reach statistical significance when they are mutually adjusted for.

## Results

### Point and lifetime prevalence rates of psychiatric disorders in school, clinical sample and total sample

The point and lifetime prevalence rates of any psychiatric disorder in the school sample were 21.89% (±4.5) and 34.09% (±5.2). When correcting these estimates with participants from the departments of child and adolescent psychiatry, the point and lifetime prevalence rates increased to 23.93% (±4.2) and 35.82% (±4.8), respectively. The figures for each diagnosis are shown in Table 1. As expected, we found large discrepancies in the lifetime prevalence rates between the sample currently in psychiatric inpatient treatment and the school sample for depressive disorders, eating disorders, anxiety disorders, non-suicidal self-injury and trauma- and stress-related disorders.

**Table 1:**
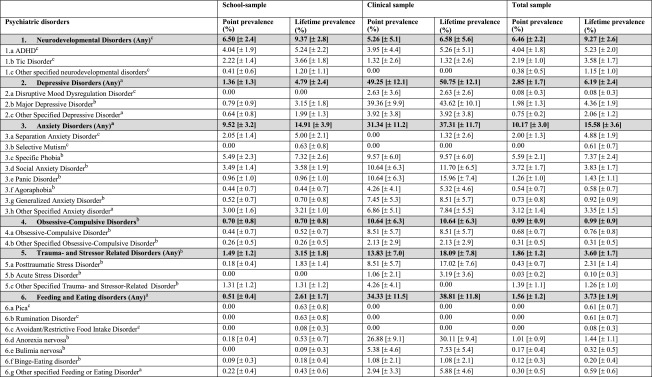

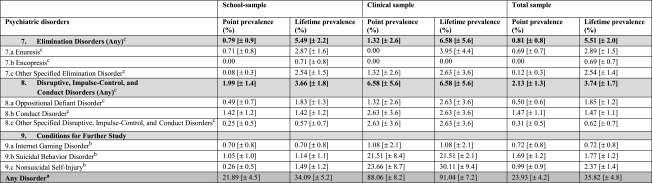
Point and lifetime prevalence rates of psychiatric disorders in adolescence: school, clinical and total sample

^a^All cases included where at both (adolescents’ and parents’) interviews were available

^b^All cases included where at least the adolescent’s interview was available

^c^All cases included where at least the parents’ interview was available

### Gender differences

Overall, there were no gender differences for the total number of lifetime diagnoses (34.41% for females and 37.95% for males, see also Table 2). However, between diagnostic groups there were some differences; for example within the group of neurodevelopmental disorders, ADHD was three times as high in boys than in girls (15.4 and 5.2%, respectively) and disruptive, impulse control and conduct disorders were six times higher in boys than in girls (7.44 and 1.26%, respectively). In contrast, lifetime prevalence rates of some internalising disorders were higher in girls than in boys: anxiety disorders were twice as high in girls than in boys (19.53 and 9.52%, respectively), trauma- and stressor-related disorders were almost four times as high in girls than in boys (4.94 and 1.30%, respectively), and feeding and eating disorders were eight times higher in girls than in boys (5.47 vs. 0.64%, respectively). Within the category “Conditions for further studies (appendix B)”, no girls were found to fulfil the criteria for Internet gaming disorder, while 2.01% of the boys fulfilled the criteria. Suicidal behaviour disorder was prevalent in 2.53% of the girls and 0.33% of the boys, and non-suicidal self-injury in 3.49% of the girls and absent in the boys, reflecting the internalising character of the disorders.

**Table 2:** Lifetime disorders (%) by sex including school and clinical sample

	Boys	Girls
1. Neurodevelopmental disorders	15.41	5.21
2. Depressive disorders	5.96	5.84
3. Anxiety disorders	9.52	19.53
4. Obsessive compulsive disorders	1.10	0.97
5. Trauma- and stressor-related disorders	1.30	4.94
6. Feeding and eating disorders	0.64	5.47
7. Elimination disorders	6.78	4.61
8. Disruptive, impulse control and conduct disorders	7.44	1.26
9.a Internet gaming disorder	2.01	0.00
9.b Suicidal behavioural disorder	0.33	2.53
9.c Non-suicidal self-injury	0.00	3.49
Any disorder	37.95	34.41

### Comorbidities

Over 40% of individuals within each diagnostic category met the criteria for another diagnostic category during their lifetime with depressive disorders and anxiety disorders being the most frequent. The highest comorbidity rates were found within the category “Conditions for further studies”. Depression and anxiety disorders were present in 87.5% and 68.8% of cases with suicidal behaviour disorder. Depression, anxiety disorders and suicidal behaviour disorder were prevalent in 84%, 60% and 44% of cases with non-suicidal self-injury. Lifetime comorbidities of all mental disorders are listed in Table 3.

**Table 3:**
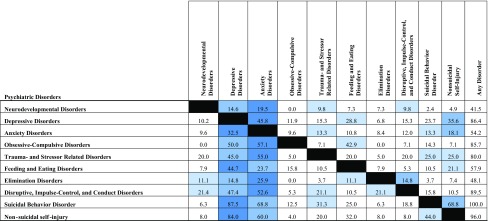
Lifetime comorbidities (%) among groups of psychiatric disorders (school and clinical sample combined)

The three highest comorbidities are highlighted in colour

Internet gaming disorder not considered due to low number of cases

### Socio-demographic, clinical and risk differences between healthy adolescents and adolescents with mental disorders in the school and clinical sample

Significant group differences were found for adolescents without lifetime mental health problems and the clinical sample with respect to sex, age, family status, chronic physical or mental illness within the family, and the history of a traumatic life event (see Table 4). The clinical sample showed a higher percentage of female patients compared to students without a full-syndrome psychiatric disorder, who were older, lived more often with a single parent, had a higher percentage of chronic physical illness and mental illness within the core family, and were more often exposed to traumatic life events such as abuse and violence. In comparison with the school sample with a full-syndrome psychiatric disorder, the clinical sample revealed a higher percentage of mental health problems within the family. No differences between the groups were found for migration background, socioeconomic status, parental employment status, comorbidity with a chronic somatic illness and negative life events. Applying logistic regression analysis, family status (*χ* ^2^ = 9.65, *p* = 0.047) and a diagnosed mental illness in the family (*χ* ^2^ = 11.13, *p* = 0.004) were significant predictors for the group membership (students with vs. without full-syndrome diagnosis vs. clinical sample), whereas age and the experience of any severe life events just failed to reach statistical significance (*p* = 0.073 and *p* = 0.066, resp.).

**Table 4:** Socio-demographic characteristics in adolescents with and without psychiatric disorders

	Students without full-syndrome psychiatric diagnosis (*n* = 199)	Students with full-syndrome psychiatric disorders (*n* = 124)	Clinical sample with full-syndrome psychiatric disorders (*n* = 61)	F or *χ* ^2^-value, *p*
Females (%)	61.3	62.9	78.7	*χ* ^2^ = 6.393, *p* = 0.041
Age (mean, SD)	13.95 (2.09)	14.16 (2.11)	14.81 (1.80)	*F* = 3.978, *p* = 0.020
Migration background				
General^a^ (%)	19.7	17.2	22.0	*χ* ^2^ = 0.644, *p* = 0.725
1st Generation^b^ (%)	1.0	3.3	0.0	*χ* ^2^ = 3.505, *p* = 0.173
2nd Generation^c^ (%)	8.3	4.1	3.4	*χ* ^2^ = 3.246, *p* = 0.197
Socioeconomic status^d^				
Low (%)	0.5	0.8	3.4	*χ* ^2^ = 6.480, *p* = 0.166
Moderate (%)	28.4	23.0	33.9	
High (%)	71.1	76.2	62.7	
Parental employment status (% employed)				
No parent	1.5	1.7	5.1	*χ* ^2^ = 4.334, *p* = 0.363
One parent	21.2	20.8	27.1	
Both parents	77.3	77.5	67.8	
Family status (%)				
Both parents	76.3	68.3	50.0	*χ* ^2^ = 16.169, *p* = 0.003
Single parent	16.0	17.5	32.8	
Patchwork	7.7	14.2	17.2	
Diagnosed chronic somatic illness^e^ (%)	14.9	15.7	27.1	*χ* ^2^ = 4.975, *p* = 0.083
Diagnosed chronic somatic illness in family (parents/siblings)^e^ (%)	22.3	30.5	50.0	*χ* ^2^ = 13.332, *p* = 0.001
Diagnosed mental illness in family (parents/siblings)^e^ (%)	4.7	8.4	25.0	*χ* ^2^ = 19.688, *p* < 0.001
Any burdensome event in life (like death, accident)^e^ (%)	39.5	52.0	45.0	*χ* ^2^ = 4.775, *p* = 0.092
Any severe event in life (like abuse, violence)^e^ (%)	7.8	14.6	25.0	*χ* ^2^ = 12.651, *p* = 0.002

^a^ Own birth place and/or birth place of one/both parents in foreign country

^b^Own birth place in foreign country

^c^Own birth place in Austria and birth place of both parents in foreign country

^d^Categorization based on Family Affluence Scale

^e^Based on adolescents’ self-reports

### GAF (global assessment of functioning) scale

The school sample and clinical sample differed with respect to global functioning with higher functioning scores in the school sample, ranging between 62.5 for non-suicidal self-injury and 82.3 for feeding and eating disorders. In the clinical sample, mean GAF scores ranged from 47.5 for neurodevelopmental disorders to 67.5 for elimination disorders. Table 5 illustrates the mean GAF scores for all psychiatric disorders, reported separately for the school and clinical samples.

**Table 5:** General assessment of functioning of mental health problems by school and clinical sample

Psychiatric disorders	GAF (mean, SD)		
	School sample	Clinical sample	School + clinical sample
Neurodevelopmental disorders	73.94 (10.86)	47.50 (23.80)	69.14 (16.93)
Depressive disorders	69.38 (13.38)	53.62 (11.76)	55.53 (12.84)
Anxiety disorders	74.52 (13.05)	54.31 (14.06)	67.24 (16.50)
Obsessive–compulsive disorders	70.50 (13.15)	54.17 (16.48)	61.59 (16.67)
Trauma- and stressor-related disorders	74.93 (13.84)	55.42 (9.41)	65.92 (15.33)
Feeding and eating disorders	82.25 (13.12)	52.76 (9.71)	57.89 (15.21)
Elimination disorders	75.00 (0.00)	67.50 (0.00)	71.25 (5.30)
Disruptive, impulse control and conduct disorders	72.50 (14.97)	60.00 (3.54)	68.93 (13.74)
Internet gaming disorder	–	–	–
Suicidal behaviour disorder	80.00 (0.00)	51.88 (13.19)	54.04 (14.84)
Non-suicidal self-injury	62.50 (0.00)	48.39 (13.25)	49.33 (13.28)
Any disorder	76.54 (11.61)	54.69 (10.56)	67.09 (15.55)
1 Disorder	78.84 (10.78)	56.09 (8.85)	73.33 (14.22)
2 Disorders	71.55 (8.46)	55.89 (6.55)	62.42 (10.70)
3 Disorders	54.83 (7.09)	58.25 (8.98)	57.46 (8.43)
>3 Disorders	–	45.31 (16.50)	45.31 (16.50)

### Service use of school-based adolescents with psychiatric conditions

In the school sample, 34.09% of the students fulfilled the diagnostic criteria for any lifetime psychiatric disorder. Within this group, 47.5% had received treatment, 8.0% with inpatient treatment and 57.3% received outpatient treatment in mental health service, psychotherapeutic or clinical-psychological treatment, and 13.9% received medication. Of the group without treatment, 18.1% expressed an interest in treatment. Mental health care utilization for each diagnostic group is listed in Table 6. Service use differs between diagnostic groups, with highest health care utilization in externalising mental health problems, including neurodevelopmental disorders (63.4%) and elimination disorders (44.4%). For internalising disorders, lower mental health care use was detected, i.e. 27.3% in anxiety disorders, 18.8% in eating disorders, 16.7% in suicidal behaviour disorders and 10% in non-suicidal self-injury.

**Table 6:** Mental health service use by lifetime mental disorders

Psychiatric disorder	Overall mental health service use (% yes)	Setting (%)^a^			Medication use (% yes)	Help wished by participant (%)^b^
		Inpatient	Outpatient	Unknown		
Neurodevelopmental disorders	63.4	5.9	70.6	23.5	17.1	22.2
Depressive disorders	41.7	6.7	60.0	33.3	5.6	23.8
Anxiety disorders	27.3	4.8	57.1	38.1	9.1	19.6
Obsessive–compulsive disorders	25.0	50.0	0.0	50.0	0.0	16.7
Trauma- and stressor-related disorders	40.0	16.7	16.7	66.7	6.7	11.1
Feeding and eating disorders	18.8	33.3	33.3	33.3	6.3	0.0
Elimination disorders	44.4	0.0	66.7	33.3	18.5	0.0
Disruptive, impulse control, and conduct disorders	38.9	0.0	71.4	26.8	0.0	18.2
Internet gaming disorder	0.0	n.a.	n.a.	n.a.	0.0	0.0
Suicidal behaviour disorder	16.7	100.0	0.0	0.0	0.0	20.0
Non-suicidal self-injury	10.0	0.0	100.0	0.0	0.0	11.1
**Any disorder**	47.5	8.0	57.3	34.7	13.9	18.1

*n.a.* not applicable

^a^% of those who use mental health services

^b^% of those who did not use mental health services

## Discussion

This is an epidemiological study of mental disorders applying DSM-5 criteria in adolescents aged between 10 and 18 years. We assessed almost 4000 adolescents for mental health problems, functional impairment and treatment seeking and included hard-to-reach groups. We used a two-stage design as it is more time efficient and can obtain samples from a broad base. The second stage, using structured clinical interview is intended to overcome limitations of exclusively using screening instruments [29].

We found an overall point prevalence of 21.89% of mental disorders in adolescent students aged 10–18 years (23.93% when correcting for a sample which cannot be reached via schools). Anxiety disorders (9.52%) were the most common followed by neurodevelopmental disorders. These prevalence rates are comparable to those found in a US nationwide epidemiological study for mental health problems in adolescents, applying DSM-IV criteria where a 30 day prevalence rate of 23.4% for any disorder and 4.5% for ADHD was found. However, the rates for anxiety disorders (14.9% vs. 11.01%), mood disorders (3.1% vs. 1.36%) and oppositional and conduct disorders (4.4% compared to 1.91%) were higher in the US sample [30].

A meta-analysis of 41 studies published between 1985 and 2012 from 27 countries concluded that the worldwide prevalence of any mental disorder in children and adolescents was 13.4%. However, the conclusion from the meta-regression analyses was that these pooled prevalence estimates are likely to be an underestimate. Prevalence estimates are influenced by the representativeness of the sample, the sample frame and the diagnostic interview used and may also differ by area and timing [12]. Another cause for higher prevalence rates in our study might be lower diagnostic requirements for some diagnoses and the incorporation of new disorders in DSM-5 compared to DSM-IV. This increase in prevalence is seen in eating disorders (3.7% vs. 1.8%) [31].

Within the category “Condition for further study” we have found lower point and lifetime prevalence rates for suicidal behaviour disorder than in German-based studies (where point and lifetime prevalence rates of suicide attempts of 1.9% and 4.1% for adolescents between 13 and 18 years were reported) [32]. Comorbidities between psychiatric disorders were common. This may be an artefact caused by the current system of classification. These empirical findings can be used to answer some of the criticisms made against DSM-5 [33]. For example, it is questionable whether non-suicidal self-injury is a stand-alone entity as it is often present in the context of depressive and anxiety disorders, borderline personality or posttraumatic stress. The same argument applies to suicidal behaviour disorder.

The differential diagnostic pattern found between genders is in line with other surveys [2, 30]. The clinical sample included a higher percentage of older female adolescents who were living with a single parent or patchwork household. The clinical group also had more chronic somatic or mental illnesses in family members (parent/sibling) and had experienced more often a severe life event such as abuse or violence. However, migration background, socioeconomic or parental employment status did not differ from adolescents without a full-syndrome diagnosis. This is in line with Rescorla et al. (2007) who found no association between YSR-scores with ethnicity, religion or geography in a worldwide survey of 24 countries [34, 35].

Single-wave, cross-sectional studies such as this are likely to underestimate the burden of disease in the general population. While around 13.3% children on average show a psychiatric diagnosis at any measurement point, almost three times this number had one or more disorders in multiple wave designs [36]. This and the fact that most mental health problems start early in life (50% before the age of 15 years and 75% before the age of 18 years) and tend to persist suggests that early interventions may be of value [37].

In our school sample, 47.5% of adolescents had engaged in treatment. This is a higher treatment rate compared with the rate of 20–25% service use by young people with mental health problems reported for the USA and European countries [38, 39]. The numbers are comparable to the study of Merikangas who found fewer than half of youth with current mental disorders receiving treatment within the mental health service and that health service use differed a lot between diagnoses with highest treatment use for neurodevelopmental disorders and lowest for internalising disorders [40]. Comorbidity and severe impairment were associated with service utilisation, especially in behaviour disorders. This is in line with a recent study of mental health care use in Germany (longitudinal BELLA study) and in the Netherlands, showing the highest treatment rates within specialist centres for attention-deficit/hyperactivity disorders [10, 41]. However, in the Netherlands in younger children, only 50% of clinically referred cases met the requirement of severe impairment. It has been discussed that many children with psychiatric symptoms and mild impairment seeking mental health care will be undiagnosed and might therefore not be treated [42]. Low service use was found for mental health problems with high egosyntonicity such as eating disorders. Our findings with treatment rates of 25% are in line with the first epidemiologic study for eating disorders in the UK applying DSM-5 criteria, where service use of 27.9% was found [43]. In contrast, the most recent data of the National Survey on Drug Use and Health reported depression as the most frequent mental health problem for receiving mental health services (56.5%) in adolescents, whereas in our sample only 36% received treatment [44].

There are several barriers to seeking treatment. The first is related to parental factors such as their recognition of the problem as an entity that can possibly be treated and the resource (time and financial resources) involved in seeking treatment [45]. Second, only a few children and adolescents meeting symptom criteria are diagnosed by health professionals [46]. Third, there is an enormous discrepancy between needs and resource availability [47]. For Austria, a shortage of inpatient, outpatient and day patient facilities by around 50% of mental health care needed and a shortage of specialists (child and adolescent psychiatrists) have been reported [48]. In 2012, there was only one child and adolescent psychiatrist available for 30,000 children and adolescents with massive regional variation. Fourth, egosyntonicity and stigma are further factors that have an impact on treatment seeking [10].

A continuum of different treatment levels, from primary to tertiary prevention, from the community to specialist health services has been proposed, and appropriate interventions should be widely available to speed initial treatment contact [49, 50]. Milder but potentially serious mental disorders might respond to measures such as psychosocial support, self-help programs and non-clinical settings in an early stage [51]. Such interventions could be developed in youth-friendly ways and disseminated through community-based channels such as educational settings in schools and be provided by Internet platforms or smart-phone applications [52]. Specialised and multidisciplinary care will be required for young people with multiple or complex needs. The tertiary system has an important part to play in the care of young people with serious mental disorders and must be strengthened. It has been stated that especially inpatient facilities are needed for young people so that they are no longer placed in adult inpatient units for mental disorders [53].

### Strengths and limitations

This study has several strengths. We did not only include a school sample, but also hard-to-reach groups such as unemployed adolescents and those hospitalized in child and adolescent departments of psychiatry. The importance of including these subgroups is supported by the fact that a higher percentage of this subgroup scored within the clinical range in the screening phase. However, the population of adolescents between 10 and 18 years also includes youths who have not been approached in our survey, such as youths who do not speak German (minor refugees, migrants), who are intellectually impaired or chronically ill and are known to have an elevated risk for mental health problems.

Second, we included a broad range (*n* = 27) of mental health problems. We assessed point and lifetime prevalence rates, sex differences in mental health problems, comorbidities, general functioning, treatment rates and request for treatment. For higher diagnostic accuracy, we used in-depth interviews performed by experts (graduated psychologists in clinical training and under permanent supervision of experienced clinicians) instead of disease-specific self-rating questionnaires.

We illustrated the gap between the prevalence of mental health problems, availability of treatment facilities and request for treatment.

Nevertheless, some limitations should be noted. Of all schools (*n* = 2547) contacted in Austria, only *n* = 428 (16.8%) agreed to participate initially, of which *n* = 75 (22.3%) declined in the subsequent stage. The response rate in the screening stage was low (47.3%) and another 57.8% dropped out in the interview stage. Non-response in the screening stage was primarily caused by the fact that many students did not bring the informed consent form signed by the parents to school and were therefore not allowed to participate. Data protection discussions that took place in the media just at the time of the data collection might also have influenced participation rates. To evaluate a possible response bias, teachers were asked to rate all students of their class (participating and not participating) regarding a few basic items. According to the teachers, students who did not participate in the screening stage were significantly more often absent from school, had lower ability to concentrate during lessons, were less likely to be well integrated in class, were more socially withdrawn in school and had more behavioural problems than students participating in the study. Although these differences reached statistical significance, the effect sizes were minimal to low. However, this might suggest that the real prevalence rates were higher. Despite great efforts made by the interviewers (at least three contact attempts, mailbox messages, text messages), a significant proportion of adolescents and parents could not have been reached for the telephone interview. Those students who participated in the interview did not significantly differ from students who could not be reached for or declined participation in the interview regarding the YSR total score, indicating that there was no selective bias in the interview stage.

However, due the low response and high dropout rate and due to the differences in the screening phase, the obtained prevalence estimates might most likely be lower than the real prevalence rates.

The use of information from multiple sources has been considered as important for obtaining a complete picture of behaviour and functioning of adolescents in epidemiologic mental health studies and reducing mono-informant information bias [11, 14]. Adolescents, parents, teachers and peers observe different aspects of behaviour and functioning and help to better understand mental health of adolescents. However, limited financial resources compel researchers to reduce the use of information sources. In our survey, we used three types of informants: Adolescents, their parents and teachers. Moreover, instead of obtaining information from different informants on the same problem, we received information from different informants about different problems: We applied the teacher’s judgment in the screening phase to rate non-participating adolescents. In stage 1, we applied self-rating questionnaires, in stage 2, we had interviewed only the adolescents for internalising problems and only parents for externalising disorders, although it has been commonly recommended to use both sources of information. So we might have lost important information and therefore could not profit from multi-informant data for comparing, contrasting and combining results obtained from different informants. This procedure might have limited the validity of the obtained diagnosis. When comparing prevalence figures across disorder and interpreting comorbidities, the fact that different information sources were used for different types of disorders should be taken into account.

Lifetime diagnoses were given retrospectively to capture mental health problems present at an earlier age. Recall bias might influence these results. Problems prevalent earlier in life might be difficult to recall for both adolescents and parents and therefore be underestimated. Additionally, transition between different diagnoses could have occured and current problems overlap previous mental health disorders.

In conclusion, this first broad nationwide and DSM-5 based study revealed a high prevalence of mental health problems in youths and low attrition to treatment despite suffering. Thus, more efforts are needed to recognize these disorders and close the gap between needs and resources for this highly vulnerable group of young people with mental health problems.

## Electronic supplementary material

Below is the link to the electronic supplementary material.

Supplementary material 1 (DOCX 25 kb)
