# Supplementary material for: Mental health problems in Austrian adolescents: a nationwide, two-stage epidemiological study applying DSM-5 criteria
Source: Eur Child Adolesc Psychiatry. 2017 May 24;26(12):1483–99. doi: 10.1007/s00787-017-0999-6 (PMC5701961; doi:10.1007/s00787-017-0999-6)
Supplement: Supplementary file 1 — Supplementary material 1 (DOCX 25 kb) [file 787_2017_999_MOESM1_ESM.docx]

**Electronic supplementary material**

Supplement to:

**Mental health problems in Austrian adolescents: A nationwide, representative, two stage epidemiological study applying DSM-5 criteria**

European Child and Adolescent Psychiatry

Gudrun Wagner, Michael Zeiler, Karin Waldherr, Julia Philipp, Stefanie Truttmann, Wolfgang Dür, Janet L. Treasure, Andreas FK Karwautz

Corresponding authors:

Gudrun Wagner, Andreas Karwautz

Department for Child and Adolescent Psychiatry, Medical University of Vienna

E-Mail: [gudrun.wagner@meduniwien.ac.at](mailto:gudrun.wagner@meduniwien.ac.at), [andreas.karwautz@meduniwien.ac.at](mailto:andreas.karwautz@meduniwien.ac.at)

**Contents**

Online Resource 1 page 2

**Online Resource 1:**  Point and Lifetime prevalence rates of psychiatric disorders of the school sample by risk status (below vs. above cut-off in the screening stage)

|  | **Point prevalence (%)** | | **Lifetime prevalence (%)** | |
| --- | --- | --- | --- | --- |
| **Psychiatric disorder** | **Below cut-off  at the screening stage** | **Above cut-off at the screening stage** | **Below cut-off  at the screening stage** | **Above cut-off at the screening stage** |
| 1. **Neurodevelopmental Disorders (Any)** ^§^ | **6.56** | **6.32** | **9.02** | **10.53** |
| 1.a ADHD^§^ | 4.10 | 3.86 | 4.92 | 6.32 |
| 1.b Tic Disorder^§^ | 2.46 | 1.41 | 3.28 | 4.93 |
| 1.c Other specified neurodevelopmental disorders^§^ | 0.00 | 1.75 | 0.82 | 2.46 |
| 1. **Depressive Disorders (Any)*** | **1.10** | **2.21** | **3.30** | **9.73** |
| 2.a Disruptive Mood Dysregulation Disorder^§^ | 0.00 | 0.00 | 0.00 | 0.00 |
| 2.b Major Depressive Disorder^#^ | 0.00 | 3.40 | 0.91 | 10.57 |
| 2.c Other Specified Depressive Disorder* | 0.74 | 0.31 | 2.22 | 1.24 |
| 1. **Anxiety Disorders (Any)*** | **8.25** | **13.72** | **13.40** | **19.91** |
| 3.a Separation Anxiety Disorder^§^ | 2.46 | 0.70 | 4.92 | 5.28 |
| 3.b Selective Mutism^§^ | 0.00 | 0.00 | 0.82 | 0.00 |
| 3.c Specific Phobia^#^ | 4.55 | 8.61 | 6.36 | 10.49 |
| 3.d Social Anxiety Disorder^#^ | 3.64 | 3.01 | 3.64 | 3.38 |
| 3.e Panic Disorder^#^ | 0.91 | 1.13 | 0.91 | 1.13 |
| 3.f Agoraphobia^#^ | 0.00 | 1.88 | 0.00 | 1.88 |
| 3.g Generalized Anxiety Disorder^#^ | 0.00 | 2.26 | 0.00 | 3.01 |
| 3.h Other Specified Anxiety disorder* | 2.96 | 3.11 | 2.96 | 4.04 |
| 1. **Obsessive-Compulsive Disorders**^#^ | **0.00** | **3.01** | **0.00** | **3.01** |
| 4.a Obsessive-Compulsive Disorder^#^ | 0.00 | 1.88 | 0.00 | 2.26 |
| 4.b Other Specified Obsessive-Compulsive Disorder^#^ | 0.00 | 1.13 | 0.00 | 1.13 |
| 1. **Trauma- and Stressor Related Disorders (Any)** ^#^ | **0.91** | **3.40** | **2.73** | **4.53** |
| 5.a Posttraumatic Stress Disorder^#^ | 0.00 | 0.75 | 1.82 | 1.89 |
| 5.b Acute Stress Disorder^#^ | 0.00 | 0.00 | 0.00 | 0.00 |
| 5.c Other Specified Trauma- and Stressor-Related Disorder^#^ | 0.91 | 2.64 | 0.91 | 2.64 |
| 1. **Feeding and Eating disorders (Any)*** | **0.00** | **2.21** | **2.06** | **4.42** |
| 6.a Pica^§^ | 0.00 | 0.00 | 0.82 | 0.00 |
| 6.b Rumination Disorder^§^ | 0.00 | 0.00 | 0.82 | 0.00 |
| 6.c Avoidant/Restrictive Food Intake Disorder^§^ | 0.00 | 0.00 | 0.00 | 0.35 |
| 6.d Anorexia nervosa^#^ | 0.00 | 0.76 | 0.00 | 2.27 |
| 6.e Bulimia nervosa^#^ | 0.00 | 0.00 | 0.00 | 0.38 |
| 6.f Binge-Eating disorder^#^ | 0.00 | 0.38 | 0.00 | 0.76 |
| 6.g Other specified Feeding or Eating Disorder* | 0.00 | 0.93 | 0.00 | 1.86 |
| 1. **Elimination Disorders (Any)** ^§^ | **0.82** | **0.70** | **4.92** | **7.39** |
| 7.a Enuresis^§^ | 0.82 | 0.35 | 2.46 | 4.23 |
| 7.b Encopresis^§^ | 0.00 | 0.00 | 0.82 | 0.35 |
| 7.c Other Specified Elimination Disorder^§^ | 0.00 | 0.35 | 2.46 | 2.82 |
| 1. **Disruptive, Impulse-Control, and Conduct Disorders (Any)** ^§^ | **1.64** | **3.16** | **3.28** | **4.91** |
| 8.a Oppositional Defiant Disorder^§^ | 0.00 | 2.11 | 1.64 | 2.46 |
| 8.b Conduct Disorder^§^ | 1.64 | 0.70 | 1.64 | 0.70 |
| 8.c Other Specified Disruptive, Impulse-Control, and Conduct Disorders^§^ | 0.00 | 1.06 | 0.00 | 2.46 |
| 1. **Conditions for Further Study (Any)** |  |  |  |  |
| 9.a Internet Gaming Disorder^#^ | **0.91** | **0.00** | **0.91** | **0.00** |
| 9.b Suicidal Behavior Disorder^#^ | **0.91** | **1.51** | **0.91** | **1.89** |
| 9.c Nonsuicidal Self-Injury^#^ | **0.00** | **1.13** | **0.91** | **3.40** |
| **Any Disorder*** | **20.62** | **26.11** | **31.96** | **41.15** |

*All cases included where at both (adolescents’ and parents’) interview were available

^#^ All cases included where at least the adolescent’s interview was available

^§^ All cases included where at least the parents’ interview was available
